# Personality Traits and Sociodemographic Variables’ Effects on Parental Burnout During the Second and Fourth COVID-19 Waves in Italian Parents

**DOI:** 10.3390/ijerph21111520

**Published:** 2024-11-15

**Authors:** Giulia Giordano, Barbara Caci, Marianna Alesi, Ambra Gentile, Sofia Burgio, Concetta Polizzi

**Affiliations:** 1 Department of Psychology, Educational Sciences and Human Movement, University of Palermo, 90128 Palermo, Italy; giulia.giordano@unipa.it (G.G.); marianna.alesi@unipa.it (M.A.); ambra.gentile01@unipa.it (A.G.); concetta.polizzi@unipa.it (C.P.); 2 WeSearch Lab—Laboratory of Behavioral Observation and Research on Human Development, University of Palermo, 90128 Palermo, Italy

**Keywords:** parental burnout, personality traits, COVID-19 pandemic, waves

## Abstract

(1) Background: Since the COVID-19 pandemic was a source of stress for families, this study aimed to investigate the influence of dispositional factors, such as personality traits and sociodemographic variables, on parental burnout among Italian parents during two waves of the COVID-19 pandemic. Therefore, the study assessed personality traits and sociodemographic variables as protective or risk factors for parental burnout levels. (2) Methods: The study consisted of two cross-sectional samples collected during the second and fourth waves of the Italian pandemic. The participants included 600 Italian parents: 245 from the second wave (average age = 37.12, SD = 2.78) and 355 from the fourth wave (average age = 36.89, SD = 3.14). The measures used were the Balance between Risks and Resources, the Personality Inventory, and a sociodemographic questionnaire. (3) Results: The *t*-test showed that parents in the fourth wave had lower parental burnout levels. Moreover, stepwise multiple linear regression revealed that sociodemographic variables did not have an effect, while significant effects of personality traits were found. Specifically, neuroticism was identified as a risk factor for parental burnout, while agreeableness and openness were identified as protective factors. (4) Conclusions: The findings indicated that similar stress levels were reported between the two waves of parents and that personality traits play a crucial role in facilitating or limiting the management of parental competencies during a risk condition.

## 1. Introduction

The COVID-19 pandemic, officially classified as a global pandemic in March 2020 [1], has undoubtedly represented a risk and “emergency” condition for the growth and lifespan development caused by the life event stressor [2], in terms of threats to the psycho-physical integrity of the organism [3].

This risk condition has led to numerous negative impacts on the mental health of children, adolescents, and adults, especially parents [4]. Among adults, many studies have found the presence of a series of symptoms related to anxiety and depression [5] and also sleep disorders, both in terms of insomnia and changes in sleep quality [6,7], emotional and behavioral dysregulation, etc.

These are implications not only due to the spread and the fear of the virus but also, and often even more, to the effects of the numerous guidelines and social restriction measures adopted (i.e., lockdown, distancing, use of masks) by governments worldwide [8]. Moreover, ample research highlighted the mental health effects of the factors mentioned above on people’s mental health, such as a significant increase in the levels of anxiety, depression, anger, alcohol use disorder, and more significant psychological distress [9]. On the other hand, some studies have investigated the impact of the pandemic on the functioning of families [10,11,12,13,14,15] and, more specifically, on the psychological health of parents [4,16]. The increase in parental stress has been underlined as has negative consequences in the parent–child relationship and, therefore, in the inadequate management of parenting competence [16,17,18]. During the COVID-19 pandemic, parents often experienced very high levels of stress in managing parental functions that could have led to parental burnout [19]; in some cases, parents showed depressive symptomatology or difficulties in managing parental competence, which could have led to several forms of mistreatment toward their children [20].

Regarding parental stress, this study assumed the Balance between Risks and Resources model [21,22] that explains parental stress as a condition of parental burnout, resulting from an imbalance between parental risks and protective factors. Parental burnout develops when parental resources are insufficient to meet the demands/risk factors, where the latter are factors that significantly increase levels of parental stress (e.g., low parental emotional intelligence, perfectionism, poor child-rearing practices, lack of support from the co-parent, and lack of social support) [21]. By contrast, resources/protection factors can be defined as factors that help to significantly decrease parental stress (e.g., parental self-compassion, high emotional intelligence, good child-rearing practices, positive co-parenting, and social support). Therefore, according to this model of parental burnout, resources are not the absence of risks but the opposite of risks. Furthermore, this model of parental stress, focused on parental burnout, differentiates between specific and common risk factors. The former, such as high parental expectations, ineffective parenting strategies, and poor co-parenting, are unique to parenting and solely predict parental burnout. In contrast, the latter, like a perfectionistic personality, inadequate stress management, and a pessimistic outlook, is related to parental and job burnout. Consequently, parents with a predominance of common risk factors are likely at risk for both types of burnout. In contrast, those with mainly parenting-specific risk factors are more susceptible exclusively to parental burnout.

Several studies have investigated the role of dispositional and sociodemographic variables, such as the parent’s age, gender, education, job or working condition, couples’ conditions, or the child’s typical/atypical development patterns, as protective or risk factors.

As regards personality, the Big Five Factors Model of personality [23] assumes five factors underlying personality, namely: extraversion, which is characterized by excitability, sociability, talkativeness, assertiveness, and high amounts of emotional expressiveness; agreeableness, which includes attributes such as trust, altruism, kindness, affection, and other prosocial behaviors; conscientiousness, which is defined by high levels of thoughtfulness, reasonable impulse control, and goal-directed behaviors; neuroticism, which is characterized by sadness, moodiness, and emotional instability; and openness (also referred to as openness to experience), which emphasizes imagination and insight. Indeed, personality traits play a crucial role among dispositional variables, as highlighted by numerous other studies that have shown the weight of personality traits in determining more or less adaptive behavioral responses to the stress caused by the pandemic with its restrictions [4,16,24,25,26,27,28,29]. Personality traits can influence parent distress, promoting or obstructing responsive parenting [30]; for example, parents with high levels of neuroticism tend to experience stressful conditions more severely and to be less responsive to the needs of their children [31]. On the other hand, people with high levels of neuroticism tend to perceive higher levels of stress in critical periods of life [32] and to have greater reactivity to stressful events, as opposed to what usually happens in people with a predominant trait of extroversion [33]. More significant stress and concerns were found in both the early and late stages of the pandemic in adults characterized by neuroticism in many European countries, including Italy, where, in contrast, high levels of extroversion appear to have played a protective role concerning the COVID-19 fear [34]. Therefore, during the COVID-19 pandemic, neuroticism represented a critical risk factor for parents’ mental health, increasing anxiety and worries related to managing parental functions. On the contrary, high levels of extraversion and emotional stability represented a protective factor for parental competence; parents with these personality traits tended to enjoy lower levels of parental stress [4,16].

Sociodemographic variables about parental burnout are studied less. Concerning gender, contrasting results emerged for parental burnout. Some studies reported that mothers are more at risk of parental burnout [35,36]. Still, as Roskam and Mikolajczak (2020) [37] noted, most studies did not examine the confounding effects of sociodemographic variables. Moreover, Le Vigouroux and Scola [38] found that younger parents were at risk of emotional exhaustion than older parents, which is confirmed by other studies [39] where parents between 33 and 42 years were more at risk of parental burnout than parents aged 42–50. This happens because young parents must raise a child and simultaneously find emotional and work stability [40]. Moreover, employed parents tend to be more at risk of parental burnout than non-occupied parents [41]. Finally, regarding the parents’ education level, higher education levels are likely related to greater awareness concerning mental health and stress, leading to greater emotional distance [42,43].

Most of these studies have considered the impact of the pandemic on parents and possible parental burnout during the first year of the pandemic (2020) and even more so during the total post-lockdown that followed the first devastating wave of the virus [44]. Pandemic waves, on the other hand, should be considered with specific attention; in fact, there may be significant differences in the conditions of psychological stress experienced by people, precisely about the specificity of the pandemic wave—e.g., about duration, perceived dangerousness, changes in the security measures taken, and the possibility of experiencing social relations [45].

Few studies have been conducted, especially in Italy, focusing on the emergency created by other waves of the virus [46,47]; specifically, the second wave (September–December 2020) was characterized by an increase in hospitalizations and deaths, even more significant than in the first wave (February–April 2020) in which the Italian government introduced a total lockdown and numerous restrictions for individual mobility, social, cultural, and work activities [48,49,50,51]. Indeed, the fourth wave occurred between October and December 2021 and was not previously investigated in any study. Still, this wave is worth considering since the vaccine’s effectiveness determined less adherence to safety behaviors and increased relational freedom, causing a new COVID-19 contagion peak. Therefore, this new contagion wave might have created experiences of stressful conditions again.

From this perspective, no previous studies have compared the emotional burden associated with the different COVID-19 waves, especially the fourth wave.

Given these considerations and starting from the idea of a possible change in parental burnout [52] between the two waves, the present study focused on stressful conditions in Italian parents’ management of parenthood, considering predictors such as sociodemographic factors and personality traits. Despite several studies carried out during COVID-19 investigating the relationship between personality traits and stress, anxiety, and coping, these rarely studied differences among waves and mainly focused on a single personality trait, such as neuroticism. Moreover, the weight of other personality traits has been little investigated [53]; in this sense, the study’s novelty focuses on comparing different pandemic stages, considering the relationships between personality traits and parental burnout.

The general goal of the current study was to investigate the association between parental burnout levels and personality traits in Italian parents during the COVID-19 pandemic. In particular, the first aim was to examine the differences in parental burnout levels between a group of parents tested during the second wave of COVID-19 in Italy (W2) and a group assessed during the fourth wave of COVID-19 (W4). Moreover, the second aim was to determine predictors for parental burnout.

Therefore, a cross-sectional design was used to compare the two groups since different participants were surveyed at each time wave. We hypothesized the following:

- Hp1: Parents in the W4 group would report lower parental burnout levels than those in the W2 group;
- Hp2: Sociodemographic variables (age, education, job, couple condition, number of sons) would have effects on parental burnout;
- Hp3: Higher levels of neuroticism would be associated with lower parental competencies;
- Hp4: Higher levels of conscientiousness, extraversion, openness, and agreeableness would be connected to higher parental competencies.

## 2. Materials and Methods

### 2.1. Participants

The sample comprised 600 Italian parents (245 from W2 and 355 from W4). In both groups, 90% of the sample was composed of mothers. Participants had at least one son aged between 4 and 17. Sociodemographic information is shown in Table 1.

Participants from the two waves, W2 and W4, were equivalent in terms of gender [χ^2^ (1599) = 0.284, *p* = 0.594], marital status [χ^2^ (4596) = 1.10, *p* = 0.894], age [t (2598) = −1.157, *p* = 0.248], and nationality [t (2598) = 1.294, *p* = 0.699].

In both waves, June–October 2020 and September–December 2021, data were collected through an online survey using a snowball sampling method; in particular, researchers sent the online survey link to colleagues and university students, who were asked to disseminate the survey through the primary means of communication and social networks (i.e., Facebook groups and WhatsApp) to reach a large number of parents with underage children, both mothers and fathers. Participation was voluntary and anonymous; all participants completed their informed consent form. Parents were asked to answer truthfully regarding their parenting experience during the pandemic, which might suggest adequate psychological support interventions. At the end of the survey, parents found a section for open comments to refer to their thoughts, feelings, and concerns about the experience of parenting during the two waves of the COVID-19 pandemic considered in the current study. The survey took about 20–25 min to be completed. In total, 800 surveys were collected, and only 600 were fully completed and valid for statistical analyses.

### 2.2. Measure

#### 2.2.1. Personality Inventory

The Personality Inventory (PI) [54] is a questionnaire comprising 20 items and five subscales; each of the four items is related to the five personality factors. For example, based on the FFM [55], the PI measures neuroticism as a tendency toward emotional instability (i.e., “I am an irritable person”), conscientiousness as the sense of duty and self-discipline (i.e., “I work hard to accomplish my work”), extraversion as a search for excitement for trendiness or sociability in terms of aggregation, assertiveness, and positive emotionality (i.e., “I like to have a lot of people around me”); openness as an experience with and intellectual curiosity for culture and experiences (i.e., “I often enjoy playing with theories and abstract ideas”); and agreeableness as cooperation and trust with others (i.e., “I’m a person who listens rather than speaks”). Each item is rated on a 5-point scale (from 1: strongly disagree to 5: strongly agree). In the current study, α coefficients of PI were 0.76 for neuroticism, 0.72 for conscientiousness, 0.71 for extraversion, 0.78 for openness, and 0.70 for agreeableness.

#### 2.2.2. The Balance Between Risks and Resources

The Balance between Risks and Resources [21] is a self-report questionnaire used to measure parental burnout. The original version was translated and adapted to the Italian context with the author’s permission. This questionnaire assesses perceptions of parental burnout through two antecedents: common and specific risk factors. The former are predictors of job and parental burnout (i.e., “When I express my emotions, I often hurt close family and/or friends”). The latter is uniquely related to parental burnout (e.g., “I don’t share good times with my children (I don’t enjoy playing with them, and/or they do not like the activities I suggest”). This was assessed through 39 bipolar rating scales encompassing 11 levels, from −5 to +5. The total score ranged between −195 and +195. Specifically, the common antecedent subscale ranged between −70 and +70, and the specific antecedent subscale ranged between −125 and +125. The total score was computed by summing the 39 items. Positive scores indicated more resources than risks, negative scores indicated more risks than resources, and 0 scores suggested that the parent had the same resources and risks. For example, “My partner denigrates me as a mother/father” is scored −5, while “My partner says that I am a good mother/father” is scored +5. The reliability values were α = 0.96 for the global scale, α = 0.89 for the common antecedent subscale, and α = 0.94 for the specific antecedent subscale.

### 2.3. Data Analysis

All statistical analyses were conducted using the software SPSS, version 26 [56]. Descriptive statistics for the observed variables were computed. Precisely, frequency, mean scores, standard deviations, and normality statistics were calculated. Skewness values lower than three and kurtosis lower than ten were sufficient to verify normal univariate distribution [57]. A Confirmatory Factor Analysis (CFA) was performed for the Italian versions of the BR2 items with the primary objective of confirming the two-factor structure proposed by Mikolajczak and Roskam [21]. The following goodness-of-fit indices were employed to determine the acceptability of the CFA model: the comparative fit index (CFI), the Tucker–Lewis index (TLI), and the root mean square error of approximation (RMSEA). Regarding the first two indices, the CFI and TLI, only values of 0.90 or greater were accepted, and values less than or equal to 0.08 for RMSEA were accepted. A robust estimation method used weighted least squares with mean and variance adjustments (WLSMV).

This multivariate analysis also assumes homogeneity of variance–covariance matrices, mainly when sample sizes differ significantly, as observed here. In this case, each group’s (wave’s) variances and covariances presented a ratio below 10:1, with the group with the most significant sample (women in W2) showing the highest variances and covariances. Thus, the alpha levels produced are conservative, and the null hypotheses can be confidently rejected [58]. To investigate Hp1, we compared the two levels of parental burnout between W2 and W4 by performing an independent *t*-test. Stepwise multiple linear regression was used to determine predictors for parental burnout and test Hp2, Hp3, and Hp4. As a first step, we weighed demographic information about parents (age, education, job type, couple type, number of sons, and sons’ age); in the second step, we added personality traits to determine the impact of both situational and dispositional predictors of parental burnout. An adjusted R2 was used. Regression coefficients and 95% confidence intervals (CIs) were computed for our model.

## 3. Results

Table 2 shows correlations and descriptive statistics for personality and parental burnout variables, including mean scores, standard deviations, skewness, and kurtosis values.

An independent sample *t*-test depicted significant differences between the two waves (2 and 4) on parental burnout levels [(M_Wave2_ = 51.1, SD_Wave2_ = 63.6; M_Wave4_ = 81.3, SD_Wave4_ = 57.3); t (1, 598) = −6.07, *p* < 0.001]. The results of a preliminary analysis for the normality of the item distribution of BR2 showed significance for all items (ranging from 0.727, *p* < 0.01 of item 9 to 961 *p* < 0.01 of item 39). The results of the CFA showed a good fit for the two-factor model of BR2 (χ^2^(501) = 5378, *p* < 0.01; CFI = 0.962; TLI = 0.942; RMSEA = 0.078). Also, the one-factor model displayed a good fit (χ^2^ (465) = 5097, *p* < 0.01, CFI = 0.958, TLI = 0.936, RMSEA = 0.075). A stepwise multiple regression analysis was used to determine the relationship between parental burnout and each personality trait, considering sociodemographic variables. In Step 1, the model including only sociodemographic variables was significant (*p* < 0.001), but it explained only 4% of the variance of parental burnout. When personality traits were included as the Step 2 variables, the model was significant [F(5588) = 21.586, *p* < 0.001)] and accounted for approximately 21% of the variance in parental burnout (R2 = 0.244 [Adj R2 = 0.213] *p* < 0.001). None of the sociodemographic variables were significant. The results showed that neuroticism affected distress in parents (β = −0.313, *p* = <0.001), and positive and significant effects were found for openness (β = 0.126, *p* = 0.002) and agreeableness (β = 0.127, *p* = <0.001). No significant effects were found for conscientiousness and extraversion. The results are shown in Table 3.

## 4. Discussion

The current study investigated parental burnout levels by analyzing personality traits and specific sociodemographic variables as risk or protective factors in two different groups, respectively, recruited during two specific and temporally distant periods of the COVID-19 pandemic (June–October 2020 as the second wave and September–December 2021 as the fourth wave).

In line with most of the studies that analyzed parental stress during the first wave of the pandemic [19,44], our data showed that Italian parents were at risk of parental burnout; in fact, most parents showed a specific difficulty in using the resources available to counter the risk factors. In line with this perspective, a study by Skjerdingstad and colleagues [59] found that parental burnout was high during the COVID-19 pandemic, with an increasing trend between the start and three months after. In general, the lack of support from school and all the different educational contexts, the sense of loneliness and isolation induced by the drastic interruption of/reduction in face-to-face relationships, and the need to reconcile smart working with childcare have certainly raised parents’ risk condition for parenting competence leading to reduced management of parenting [60]. Indeed, parents were faced with a reality they had never experienced, a source of a thousand fears not only and not so much for themselves but above all for their children: first of all, the fear for their physical safety, as they reported in an open comment at the end of the survey on their experience as parents during the pandemic (i.e., I am afraid of getting infected and infecting my children….you can die….how are we going to do it?…and if we end up in the hospital?…we are alone…).

Specifically regarding the Italian fourth wave, the hypothesis of lower levels of parental burnout (Hp1) was confirmed by average scores in the BR total and the specific antecedents. This might be traced back to getting used to a daily life constantly crossed by the pandemic and returning to relationships despite the persistence of some restrictions (i.e., facial mask usage). Nonetheless, regarding lower parental burnout levels, our findings highlighted that scores were lower but not as low as hypothesized. This suggests that parents still have difficulties managing parenting functions, living in a new risk condition determined by the accumulated tiredness and uncertainties regarding the future. In short, the fourth wave might have once again created a relational rift, certainly putting much stress on families with children, especially if they are minors. Considering that mass media referred to the fourth wave as a psychological emergency, the most damaging effects were on people’s psychological well-being [61].

Exploring the relationships between parental burnout and personality traits and their role as protective or risk factors was partially confirmed since we found significant effects only concerning three personality traits. In particular, neuroticism was found to predict parental burnout as a risk factor, while agreeableness and openness influenced protective factors, regardless of the wave considered. Previous studies reported that the dispositional variable might play a decisive role in managing difficulties in carrying out parental functions due to the COVID-19 pandemic [4,16]. Similarly to our study, other studies showed that neuroticism is correlated with parents’ perception of themselves as having fewer resources to face the challenges related to the parental role. In this sense, parents characterized by neuroticism as a predominant personality trait seem to experience higher levels of anxiety, fear of COVID-19, insecurity, and emotional dysregulation during the pandemic [11]. Indeed, high levels of neuroticism might result in higher risk perception, higher sensitivity to stimuli, a general prevalence of negative emotions, and higher difficulty activating coping in the face of stressful events [61,62]. In the case of parents, a high level of neuroticism might determine rigid and intrusive parenting styles, increasing the risk of burnout. Conversely, agreeableness and openness [21,38] might act as protectors as they help in employing new ways of behaving and ideas, encouraging an open discussion about emotions, and looking for external emotional support [63,64]. Even if the results of the first model of the stepwise regression analyses evidenced a significant effect of sociodemographic variables, in the final model, the results were not significant. Such results could be due to an indirect effect of these variables in influencing parental burnout.

To sum up, the results of this study suggested that three personality traits, neuroticism, agreeableness, and openness, were critical factors in managing stress in daily parenting concerns and showed how living conditions caused by COVID-19 strongly increased the risk of family burnout, orienting a low perception of themselves as parents. Despite the lower parental burnout levels in W4, the persistence of high levels of burnout risk suggests that parents still have difficulties managing their parenting functions, especially parents with neurotic personalities. On the other hand, openness and agreeableness might lead parents to accept changes, which could have led to better stress management.

In addition, the study fills the literature gap on different parental burnout levels and the relationship between personality traits and parental burnout. If the results of this study are encouraging, some limits must be acknowledged. First, our comparison between W2 and W4 on parental burnout is based on a cross-sectional research design, and no causal associations could have been tested. A longitudinal study could have allowed for assessing changes in parental burnout over time. However, a longitudinal study would have been complex since an extensive population survey was used.

Moreover, data were assessed through self-reports, and the choice of data depended on the pandemic conditions. However, future studies could include other reporting measures, such as interviews, direct observations, and focus groups, to determine the pattern of behaviors better.

## 5. Conclusions

The study offers some essential suggestions for future research and mental health protection interventions in risk conditions like the one created by the COVID-19 pandemic. In terms of research, it appears to be very important to increase longitudinal studies showing long-term effects on populations affected by a pandemic, given that many aspects of people’s psychological fragility persist for a long time. From this point of view, in pandemic conditions, people’s stress, in our specific case, that of parents, may not diminish significantly over time; in this sense, psychological and psycho-educational support interventions should be thought of as accompanying people throughout the pandemic, even when the most critical time of the pandemic seems far off.

In addition, the study suggests that these interventions should carefully consider not just the sociodemographic characteristics but also the personality traits of the persons in charge, and making them aware of how much a specific psychological functioning is oriented in a neurotic sense rather than in an open-minded or amicable sense can counteract or facilitate the management of the difficulties posed by the pandemic. Therefore, the research should focus more on exploring the relationship between personality traits and coping styles.

In line with previous studies, activating psychological intervention in risky situations (such as the pandemic) to enhance emotional competency can support parents in emotional regulation, facilitating a reduction in neuroticism’s negative influence [65]. Therefore, personality factors should be considered, especially in risk conditions, to understand parental burnout.

## Figures and Tables

**Table 1:** Sociodemographic information for W2 and W4 groups.

		Waves	
		W2	W4
Variables		% or *n*	% or *n*
Parent	Mothers Fathers	91.4% 8.6%	90.1% 9.9%
Parents’ age	<36	38	38
	36–45	133	194
	>45	74	123
Education	Primary school diploma	1	5
	Middle school diploma	16	36
	Professional school diploma	23	57
	High school	78	126
	Master’s degree	92	116
	PhD or specialization	35	15
Job	Housewife	29	47
	Student	9	8
	Employee	2	32
	Worker	95	67
	Manager	12	2
	Artisan	3	1
	Shop keeper	7	7
	Freelancer	43	23
	Teacher	32	150
	Unemployed	12	11
	Job seeker	0	3
	Retired	1	0
	Other	0	4
Couple condition	Married	204	301
	Cohabitant	21	28
	Separated	18	22
	Widowed	0	1
	Single parent	2	3
Number of sons	1 2 3 >3	62 142 36 5	93 206 50 6

**Table 2:** Correlation between personality factors and BR2.

Variables	1	2	3	4	5	6	7	8
1. Neuroticism	-							
2. Consciousness	−0.227 **	-						
3. Openness	−0.205 **	0.256 **	-					
4. Extraversion	−0.086 *	0.111 **	0.136 **	-				
5. Agreeableness	0.096 *	0.047	−0.063	−0.057	-			
6. Common antecedents	−0.439 **	0.157 **	0.215 **	0.139 **	0.059	-		
7. Specific antecedents	−0.272 **	0.160 **	0.175 **	0.085 *	0.113 **	0.812 **	-	
8. BR total	−0.348 **	0.166 **	0.198 **	0.111 *	0.096 *	0.921 **	0.974 **	-
M	9.17	15.58	12.75	12.20	13.41	21.01	44.99	68.99
SD	2.92	2.54	2.55	1.83	2.68	23.41	39.30	61.70
Skewness	0.33	−0.60	−0.07	−0.21	−0.16	−0.61	−1.14	−1.00
Kurtosis	−0.08	0.56	0.17	2.11	0.23	0.18	1.38	0.199

Note. * Correlation is significant at *p* < 0.05; ** Correlation is significant at *p* < 0.01.

**Table 3:** The results of Step 2 of the linear regression model predicting parent burnout.

Predictor	b	SE	β	t	*p*
(Constant)	22.948	29.729	0.057	1.113	0.226
Age	0.586	2.308	0.012	0.254	0.800
Education	−2.614	2.278	−0.047	−1.148	0.252
Job	1.430	0.800	0.072	1.787	0.074
Couple condition	−12.693	3.513	−0.135	−3.613	0.081
Number of sons	−2.436	3.442	−0.0.27	−7.08	0.479
Neuroticism	−6.614	0.829	−0.313	−7.977	<0.001
Conscientiousness	1.077	0.961	0.044	1.121	0.263
Openness	3.048	0.999	0.126	3.050	0.002
Extraversion	2.309	1.273	0.069	1.813	0.070
Agreeableness	2.927	0.871	0.127	3.358	0.001

## Data Availability

Data are available upon request to the corresponding author.
